# Supplementary figures and images for: Quantitation of ethanol in UTI assay for volatile organic compound detection by electronic nose using the validated headspace GC-MS method
Source: PLoS One. 2022 Oct 6;17(10):e0275517. doi: 10.1371/journal.pone.0275517 (PMC9536638; doi:10.1371/journal.pone.0275517)

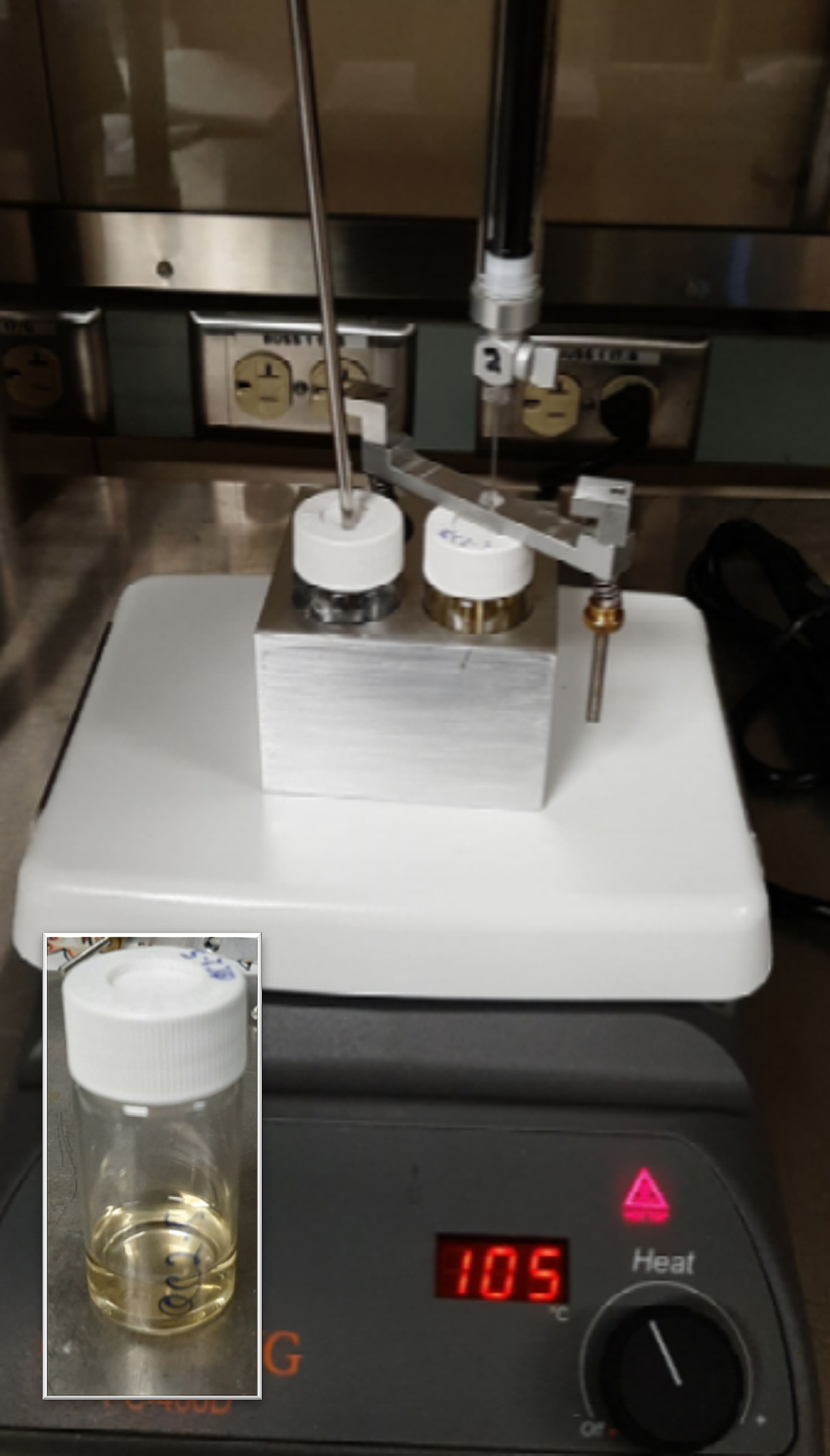

Supplement: S1 Fig — The real temperature was measured in an adjacent water vial by a digital thermometer. Inset: Urine sample in a septa-top vial for headspace analysis. (TIF) [file pone.0275517.s001.tif]

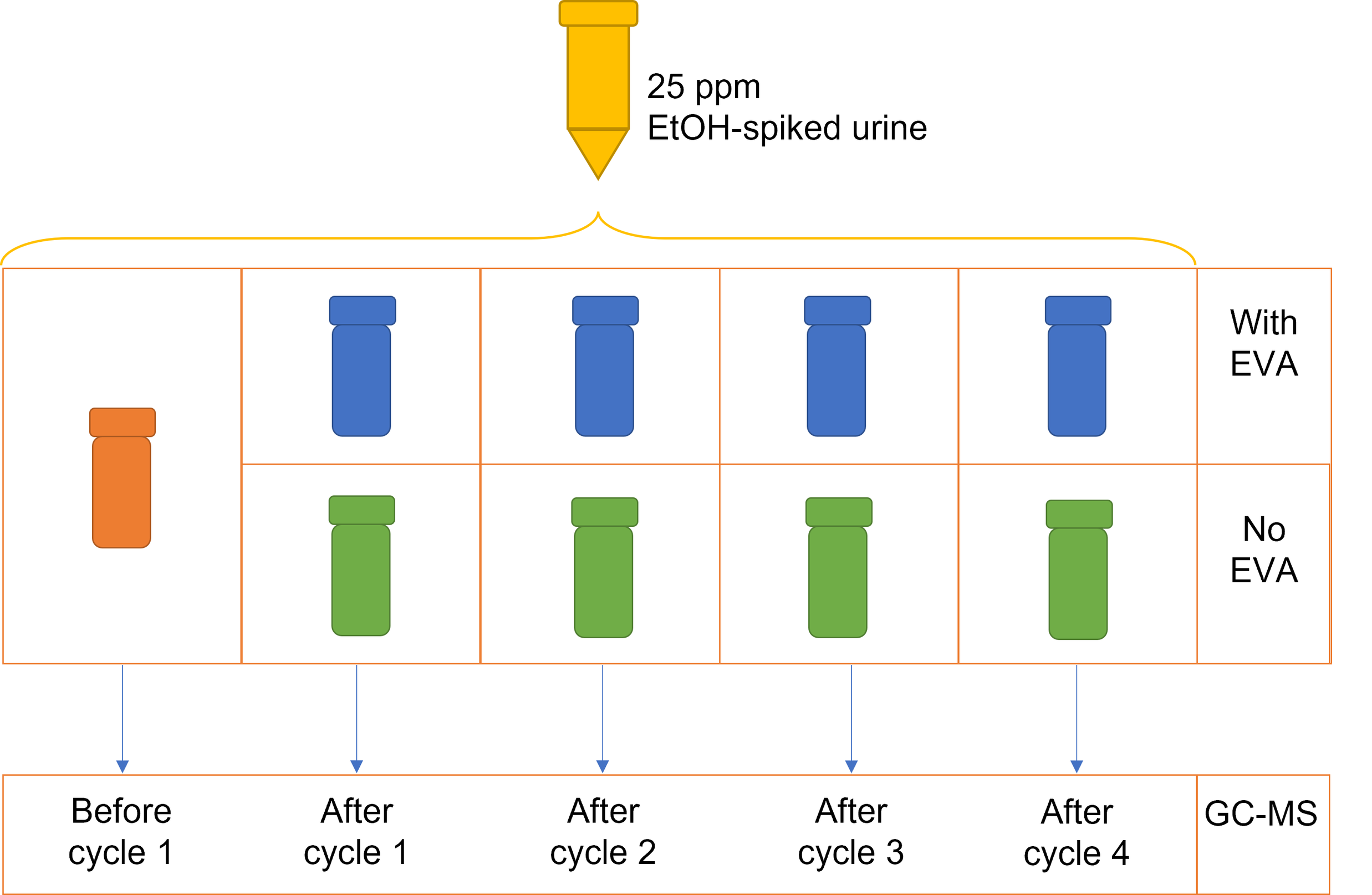

Supplement: S2 Fig — (TIF) [file pone.0275517.s002.tif]

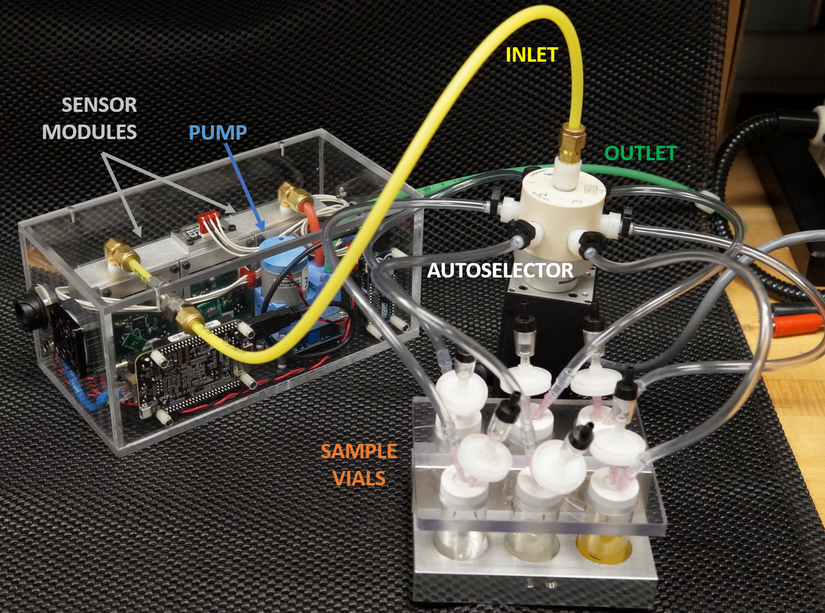

Supplement: S3 Fig — (TIF) [file pone.0275517.s003.tif]
