# Supplementary material for: Quantitation of ethanol in UTI assay for volatile organic compound detection by electronic nose using the validated headspace GC-MS method
Source: PLoS One. 2022 Oct 6;17(10):e0275517. doi: 10.1371/journal.pone.0275517 (PMC9536638; doi:10.1371/journal.pone.0275517)
Supplement: Attachment — Submitted filename: PONE-D-21-22287 (1).pdf [file pone.0275517.s004.pdf]

PLOS ONE

Quantitation of ethanol in UTI assay for volatile organic compound detection by electronic nose using the validated headspace GC-MS method

--Manuscript Draft--

**Manuscript Number:**	PONE-D-21-22287
**Article Type:**	Research Article
**Full Title:**	Quantitation of ethanol in UTI assay for volatile organic compound detection by electronic nose using the validated headspace GC-MS method
**Short Title:**	Quantitation of ethanol for volatile organic compound detection by electronic nose using headspace GC-MS
**Corresponding Author:**	Aminat Adebiyi, Ph.D.<br>University of Southern California<br>Los Angeles, CA UNITED STATES
**Keywords:**	gas chromatography mass spectrometry; method development; ethanol; electronic nose; gas sensors; Artificial neural network; urinary tract infection; Escherichia coli
**Abstract:**	Disease detection through gas analysis has long been the topic of many studies because of its potential as a rapid diagnostic technique. In particular, the pathogens that cause urinary tract infection (UTI) have been shown to generate different profiles of volatile organic compounds, thus enabling the discrimination of the causative agents using an electronic nose. While past studies have performed data collection on either agar culture or jellified urine culture, this study measures the headspace volume of liquid urine culture samples. Evaporation of the liquid and the presence of background compounds could introduce variability to the collected data. Therefore, a headspace gas chromatography-mass spectrometry method was developed and validated for quantitating ethanol in the headspace of the urine samples. By leveraging the new method to characterize the sample stability during e-nose measurement, it was revealed that ethanol concentration dropped more than 15% after only three measurement cycles, which equal 30 minutes for this study. It was further shown that by using only data within the first three cycles, better accuracies for between-day classification were achieved, which was 73.7% and 97.0%, compared to using data from within the first nine cycles, which resulted in 65.0% and 81.1% accuracies. Therefore, the newly developed method provides better quality control for data collection, paving ways for the future establishment of a training data library for UTI.
**Order of Authors:**	Nam Than, MS
	Zamri Chik
	Amy Bowers
	Luisa Bozano
	Aminat Adebiyi
**Additional Information:**	
**Question**	**Response**
**Financial Disclosure**<br><br>Enter a financial disclosure statement that describes the sources of funding for the work included in this submission. Review the submission guidelines for detailed requirements. View published research articles from *PLOS ONE* for specific examples.	The author(s) received no specific funding for this work.

Powered by Editorial Manager® and ProduXion Manager® from Aries Systems Corporation

This statement is required for submission and **will appear in the published article** if the submission is accepted. Please make sure it is accurate.<br><br>**Unfunded studies**<br>Enter: *The author(s) received no specific funding for this work.*<br><br>**Funded studies**<br>Enter a statement with the following details:<br>• Initials of the authors who received each award<br>• Grant numbers awarded to each author<br>• The full name of each funder<br>• URL of each funder website<br>• Did the sponsors or funders play any role in the study design, data collection and analysis, decision to publish, or preparation of the manuscript?<br>• **NO** - Include this sentence at the end of your statement: *The funders had no role in study design, data collection and analysis, decision to publish, or preparation of the manuscript.*<br>• **YES** - Specify the role(s) played.<br><br>* typeset	
**Competing Interests**<br><br>Use the instructions below to enter a competing interest statement for this submission. On behalf of all authors, disclose any competing interests that could be perceived to bias this work—acknowledging all financial support and any other relevant financial or non-financial competing interests.<br><br>This statement is required for submission and **will appear in the published article** if the submission is accepted. Please make sure it is accurate and that any funding sources listed in your Funding Information later in the submission form are also declared in your Financial Disclosure statement.<br><br>View published research articles from *PLOS ONE* for specific examples.	The authors have declared that no competing interests exist.

**NO authors have competing interests**<br><br>Enter: *The authors have declared that no competing interests exist.*<br><br>**Authors with competing interests**<br><br>Enter competing interest details beginning with this statement:<br><br>*I have read the journal's policy and the authors of this manuscript have the following competing interests: [insert competing interests here]*<br><br>* typeset	
**Ethics Statement**<br><br>Enter an ethics statement for this submission. This statement is required if the study involved:<br><br>• Human participants<br>• Human specimens or tissue<br>• Vertebrate animals or cephalopods<br>• Vertebrate embryos or tissues<br>• Field research<br><br>Write "N/A" if the submission does not require an ethics statement.<br><br>General guidance is provided below. Consult the submission guidelines for detailed instructions. **Make sure that all information entered here is included in the Methods section of the manuscript.**	N/A

Powered by Editorial Manager® and ProduXion Manager® from Aries Systems Corporation

**Format for specific study types**<br><br>**Human Subject Research (involving human participants and/or tissue)**<br>• Give the name of the institutional review board or ethics committee that approved the study<br>• Include the approval number and/or a statement indicating approval of this research<br>• Indicate the form of consent obtained (written/oral) or the reason that consent was not obtained (e.g. the data were analyzed anonymously)<br><br>**Animal Research (involving vertebrate animals, embryos or tissues)**<br>• Provide the name of the Institutional Animal Care and Use Committee (IACUC) or other relevant ethics board that reviewed the study protocol, and indicate whether they approved this research or granted a formal waiver of ethical approval<br>• Include an approval number if one was obtained<br>• If the study involved *non-human primates*, add *additional details* about animal welfare and steps taken to ameliorate suffering<br>• If anesthesia, euthanasia, or any kind of animal sacrifice is part of the study, include briefly which substances and/or methods were applied<br><br>**Field Research**<br><br>Include the following details if this study involves the collection of plant, animal, or other materials from a natural setting:<br>• Field permit number<br>• Name of the institution or relevant body that granted permission	
**Data Availability**<br><br>Authors are required to make all data underlying the findings described fully available, without restriction, and from the time of publication. PLOS allows rare exceptions to address legal and ethical concerns. See the PLOS Data Policy and FAQ for detailed information.	Yes - all data are fully available without restriction

A Data Availability Statement describing where the data can be found is required at submission. Your answers to this question constitute the Data Availability Statement and **will be published in the article**, if accepted.<br><br>**Important:** Stating 'data available on request from the author' is not sufficient. If your data are only available upon request, select 'No' for the first question and explain your exceptional situation in the text box.<br><br>Do the authors confirm that all data underlying the findings described in their manuscript are fully available without restriction?	
**Describe where the data may be found in full sentences. If you are copying our sample text, replace any instances of XXX with the appropriate details.**<br><br>• If the data are **held or will be held in a public repository**, include URLs, accession numbers or DOIs. If this information will only be available after acceptance, indicate this by ticking the box below. For example: *All XXX files are available from the XXX database (accession number(s) XXX, XXX.*).<br>• If the data are all contained **within the manuscript and/or Supporting Information files**, enter the following: *All relevant data are within the manuscript and its Supporting Information files.*<br>• If neither of these applies but you are able to provide **details of access elsewhere**, with or without limitations, please do so. For example:<br><br>*Data cannot be shared publicly because of [XXX]. Data are available from the XXX Institutional Data Access / Ethics Committee (contact via XXX) for researchers who meet the criteria for access to confidential data.*<br><br>*The data underlying the results presented in the study are available from (include the name of the third party*	Data access are available upon request. GC-MS data are available at https://app.box.com/folder/125090388794 and Electronic nose EVA data at https://app.box.com/folder/113625571232

and contact information or URL).<br>• This text is appropriate if the data are owned by a third party and authors do not have permission to share the data.<br><br>* typeset	
Additional data availability information:	

Powered by Editorial Manager® and ProduXion Manager® from Aries Systems Corporation

Manuscript Click here to access/download;Manuscript;Plos1_Manuscript_210803.docx

QUANTITATION OF ETHANOL IN UTI ASSAY FOR VOLATILE ORGANIC COMPOUND DETECTION BY ELECTRONIC NOSE USING THE VALIDATED HEADSPACE GC-MS METHOD

Nam Than[1¶,#a] , Zamri Chik[2¶], Amy Bowers[3], Luisa Bozano[3], and Aminat Adebiyi[3*]

[1] Department of Biomedical Engineering, San Jose State University, San Jose, California 95192, USA

[2] Department of Pharmacology, Faculty of Medicine, University of Malaya, 50603 Kuala Lumpur, Malaysia

[3] IBM Almaden Research Center, San Jose, California 95120, USA

[#a] Current Address: Department of Biomedical Engineering, The University of Texas at Austin, Austin, Texas 78712, USA

* Corresponding author
E-mail: aminat.adebiyi@ibm.com (AA)

[¶] These authors contributed equally to this work

Abstract

Disease detection through gas analysis has long been the topic of many studies because of its potential as a rapid diagnostic technique. In particular, the pathogens that cause urinary tract infection (UTI) have been shown to generate different profiles of volatile organic compounds, thus enabling the discrimination of the causative agents using an electronic nose. While past studies have performed data collection on either agar culture or jellified urine culture, this study measures the headspace volume of liquid urine culture samples. Evaporation of the liquid and the presence of background compounds could introduce variability to the collected data. Therefore, a headspace gas chromatography-mass spectrometry method was developed and validated for quantitating ethanol in the headspace of the urine samples. By leveraging the new method to characterize the sample stability during e-nose measurement, it was revealed that ethanol concentration dropped more than 15% after only three measurement cycles, which equal 30 minutes for this study. It was further shown that by using only data within the first three cycles, better accuracies for between-day classification were achieved, which was 73.7% and 97.0%, compared to using data from within the first nine cycles, which resulted in 65.0% and 81.1% accuracies. Therefore, the newly developed method provides better quality control for data collection, paving ways for the future establishment of a training data library for UTI.

Introduction

It is widely known that different types of disease can influence the pattern of volatile organic biomarkers released in exhaled gas or waste material in patients [1]. As an example, these fingerprint volatile organic compound (VOC) profiles have enabled the detection and monitoring of chronic kidney disease and its progression by gas chromatography (GC) [2]. While breath and fecal gas analyses, as used in the kidney study, are popular applications of these techniques, headspace analysis of fluid such as urine can also yield remarkable detection capability, especially in the presence of pathogens [3,4]. Therefore, these techniques have great potential as a diagnostic method for infectious diseases, stemming from the fact that different bacterial species can generate different profiles of VOCs. Bos et al. systematically reviewed the literature for the headspace VOC of six bacterial species and found that while they share some compounds, they form different patterns as some compounds are uniquely produced by only certain species [5]. Even for shared VOCs, another group was able to show, by utilizing Selected Ion Flow Tube Mass Spectrometry (SIFT-MS), that the headspace concentration is also dependent on the microorganism identity [6]. In this age of antibiotic resistance, it is critical to perform a diagnosis of UTI in real-time to minimize broad-spectrum antibiotics use, and E-nose has great potential for such an application.

Static headspace gas chromatography is a technique used for the concentration analysis of volatile organic compounds. This technique is relatively simple and can provide sensitivity that is similar to dynamic purge and trap analysis. The popularity of this technique has grown and gained worldwide acceptance for analyses of alcohol in blood, urines, and other biological samples, as well as residual solvents in pharmaceutical products. Sample matrices like blood, plastic, and cosmetics contain high molecular

weights, non-volatile material that can remain in the GC system and result in poor analytical performance. Many laboratory analysts use extensive sample preparation techniques to extract and concentrate the compounds of interest from this unwanted non-volatile material. These extraction and concentration techniques can become time-consuming and costly. Static headspace analysis avoids this time and cost by directly sampling the volatile headspace from the container in which the sample is placed.

While state-of-the-art methods such as GC-MS and SIFT-MS are useful in characterizing VOC profiles, they are usually expensive, non-portable, and time-consuming in terms of sample preparation. As alternatives, there are a plethora of studies using commercially available sensors for the same purpose, which lead to a technology called Electronic Nose (e-nose). An e-nose is essentially a system of gas sensors with pattern recognition capability to detect diseases. An e-nose must consist of appropriate hardware and software. Hardware refers to the type of sensors, most commercially available being Metal Oxide (MOX), and software refers to the analytical method used, usually in the form of a machine learning algorithm. Table 1 summarizes some different combinations of sensors and analytical methods used in past studies. Noticeably, a minimum of six sensors with an artificial neural network (ANN) model was enough to yield a high prediction accuracy, albeit a small number of labels of two.

Table 1. Literature Survey of Sensors and Classification Models for E-Nose Studies

Study	Sensors	Model	Labels	Medium	Average Accuracy

Craven (1997) [7]	6 MOX	Multi-layer perceptron and Linear discriminant analysis	4	Nutrient broth	82.2%
Gibson et al. (1997) [8]	14 Conductive polymers	Multi-layer perceptron	13	Nutrient agar	89.7%
Gardner et al. (2000) [9]	6 MOX	ANN with backpropagation	2	Blood agar then nutrient broth	96.0%
Pavlou et al. (2002) [10]	14 Conductive polymers	Genetic algorithm and backpropagation neural network	4	Agar, Brain heart infusion, cooked meat broth	95.0%
Roine et al. (2014) [4]	Commercial ion mobility spectrometer-	Linear discriminant analysis and	5	Normal urine made	83.9%

	based e-nose and 6 MOX	logistic regression		into an agar	
Asimakopoulos (2014) [11]	8 metalloporphyrin-coated sensors	Supervised Partial Least Square–Discriminant Analysis	2	Urine	84.8%

While there are many more e-nose technologies, Table 1 is limited to those used in urinary pathogen detection. Most of these studies were conducted using non-physiological media such as nutrient broth, agar, or urine made into an agar. The only urine-based study in the table was tested for prostate cancer. We envisioned that point-of-care prediction of UTI should be performed directly on a patient's urine sample. This lack of e-nose studies on liquid urine presents a need for urine-based tests for UTI. Also, *Escherichia coli* is known to be the most prevalent cause of UTI [12]. Therefore, a simple *in vitro* UTI model could be created by artificially infecting commercially available donated urine with pathogenic *E. coli* to test the e-nose system.

In this paper, we introduce the Electronic Volatile Analyzer (EVA), a rapid, gas sensing Internet of Things (IoT) platform under development at IBM Research, Almaden – designed to deliver classification results under two minutes. The main objective of this study was to demonstrate the utility of EVA in differentiating between a nonpathogenic (K12) and a uropathogenic (UPEC) strain of *E. coli* in inoculated urine in conjunction with

using a validated GC-MS method to quantify ethanol as one of the VOCs driving *E. coli* strain differentiation.

However, urine samples are known to degrade over time [13]. Minimization of the variability in the collected data caused by evaporation and sample degradation must be achieved before confidently and correctly establishing a library of training data for future real-time, point-of-care prediction of UTI. Therefore, a GC-MS method was needed for characterizing the headspace samples so that decisions can be made regarding the optimal measurement time, following by the exclusion of examples that do not genuinely represent the intended labels, which could poison the analytical model.

There was little evidence in the literature that there exists an exact method for the determination of ethanol concentration in urine using headspace [illegible]C-MS. Table 2 summarizes the literature survey. Also, Tangerman argued that the headspace technique is notorious for requiring a substantial amount of labor and volumes of the biological specimen while being less sensitive [14]. However, the headspace method more closely represents the approach done with the e-noses. Therefore, a new method for the quantitation of ethanol concentration in the headspace volume of urine samples inoculated with *E. coli* was developed and validated in this study. Finally, it was used to help improve the classification accuracy of the EVA.

Table 2. Literature Survey of GC Methods for the Quantitation of Ethanol in Biological Matrices

Study	Analyte	Sample Matrix	Method

Mihretu *et al.* (2020) [15]	Ethanol	Blood	Headspace GC-FID (Flame ionization detector)
Chun *et al.* (2016) [16]	Alcohols	Brain tissue	Headspace GC-FID
Xiao et al. (2014) [17]	Ethanol	Blood	Headspace GC-MS
Kristoffersen *et al.* (2006) [18]	Ethanol	Whole blood and plasma	Headspace GC-FID
Smith *et al.* (1999) [19]	Alcohols	Urine	Headspace SIFT-MS
Tangerman (1997) [14]	Ethanol	Whole blood, serum, urine, fecal supernatants	Direct Injection GC-MS

There are also portable systems capable of detecting ethanol, such as the zNose and GC-IMS, which are gas sensors coupled with a GC column. However, we are interested in quantifying ethanol for assisting with establishing a working protocol for the EVA. Therefore, a GC-MS method will be developed and validated for ethanol quantification in urine.

Materials and methods

The IBM Electronic Volatile Analyzer™

The EVA (Fig 1) an electronic nose under development at IBM Research Almaden. The platform consists of a modular design with each gas sensor mounted on its own

sensor module which is a printed circuit board of common design carrying an integrated microcontroller and the circuitry needed to operate the sensor. The sensor modules communicate via I2C protocol with a central hub (BeagleBone Black), which is a single-board computer that orchestrates the modules and processes the multi-sensorial output. Table 3 describes the set of six commercial MOX gas sensors used to collect the measurements described in the following sections. The selection of the sensor portfolio was based on the indications of their respective manufacturers regarding the nominal target gases of each sensor. These sensors together target CO, H_2, ethanol, methane, ammonia, H_2S, and unspecified combustible gases. Consequently, the as-selected sensor array was expected to respond to a variety of volatile molecules, including alcohols, hydrocarbons, ammonia, methane, as a well as a wide range of volatile organic compounds (VOC), with a lower limit of detection at parts per million (ppm) level.

Fig 1. The Electronic Volatile Analyzer (EVA). (A) Sensor array prototype with parts labeled. (B) A block diagram of key components of the EVA.

Table 3. MOX Sensors for the Electronic Volatile Analyzer

Sensor	Targeted gases	Commercial application	Manufacturer
GGS2330	CO, H_2, ethanol	Wide range applications	Umwelt Sensor Technik, Germany
GGS1330	Hydrocarbon, combustible gases	Gas leak detection	Umwelt Sensor Technik, Germany

TGS2611	Methane	Gas leak detection	Figaro USA, Inc., USA
TGS2602	VOCs and odorous gases such as ammonia and H_2S	Indoor air quality monitoring	Figaro USA, Inc., USA
TGS2600	H_2, ethanol, air pollutants	Indoor air quality monitoring	Figaro USA, Inc., USA
TGS8100	H2, ethanol, air pollutants	Indoor air quality monitoring	Figaro USA, Inc., USA

Each MOX sensor was operated using an individualized multi-step, periodic voltage profile applied to the heating element, which resulted in stepwise modulation of the device temperature (Fig 2). Modulation of the temperature of MOX sensing elements is a well-known technique that can be used to enhance and tune the dependence of the sensing element resistance to the surrounding environment. The heater voltage profiles applied to the EVA sensors were optimized to maximize the sensitivity of each sensor with respect to a subset of target VOC, as well as to improve response orthogonality. Although the duration and amplitude of the individual waveforms was adjusted independently for each sensor, all waveforms were synchronized to a period of 80 s to simplify the handling and processing of the sensor array outputs. The resistance of each MOX sensing element was monitored at a fixed voltage and a rate of 10 Hz. During operation, a constant flow of 150 ± 10 sccm was established by means of a small pump, drawing air from the environment in a continuous fashion while also collecting the headspace of the sample of interest. The vapor-carrying flow was directed towards an

enclosed chamber containing the six MOX gas sensors for the exposure and detection to take place.

Fig 2. Example of a temperature profile modulation for MOX sensors for IBM EVA™: (i) periodic waveform of heater voltage, expressed as a percentage of the maximum operating voltage recommended by the sensor manufacturer; (ii) corresponding variations in MOX sensor resistance under constant environment.

Chemicals and Reagents

Normal Human Urine was purchased from UTAK Laboratories Inc., USA. The vendor obtained consent from the donors, who were healthy and drug-free. Two strains of *E. coli* were purchased from the American Type Culture Collection (ATCC). A nonpathogenic strain (ATCC 29425) is designated as K12, and the other is a uropathogenic strain (ATCC 700928) designated as CFT073. They are, henceforth, referred to as K12 and UPEC, respectively. Bacterial growth medium was prepared from Tryptic Soy Broth (TSB) powder (BD Bacto). For GC-MS, HPLC-grade ethanol (EtOH) and isopropyl alcohol (IPA) were obtained from Sigma Aldrich, USA.

Preparation of EVA Samples and GC-MS Reagents

After thawing, the urine bottle was vigorously shaken and aliquoted into multiple 50-ml centrifuge tubes. Upon usage, the tubes were centrifuged at 15,000 rpm for 15 minutes to concentrate any visible sediments, which were then removed by filtration. The resultant urine was named filtered normal urine or fNU.

The TSB medium was mixed and autoclaved using the standard method printed on its bottle. The powder form of the bacteria was resuspended in liquid TSB. Each suspension was then streaked on a Tryptic Soy Agar using standard aseptic techniques. The presence of many colonies after 24 hours of incubation at 37°C indicated bacterial viability. The handling of the UPEC was carried out by BSL-2 trained personnel under a BSL-2 cabinet. The agars with colonies were stored at 4-7°C and re-streaked every two weeks to keep the cell line fresh.

One colony of each *E. coli* strain was aseptically inoculated in separate tubes of 10 ml of fNU. The cultures were incubated for 24 hours before being filtered using the same procedure for fNU, except the centrifugation speed was 5000 rpm to avoid the destruction of the bacterial cells that can inadvertently introduce unwanted substances to the supernatant. Five milliliters of the supernatant was transferred to a septa-top vial and kept in the refrigerator at 4-7°C until analysis.

A stock solution for EtOH at a 1/100 dilution was prepared by adding 1 ml of pure, 200-proof ethanol in 9 ml of Millipore water in a tube labeled 1/10 EtOH. After vortexing, 1 ml of the 1/10 EtOH was added to 9 ml of Millipore water in a tube labeled 1/100 EtOH and vortexed. The 1/100 solution of IPA was prepared in a similar manner from a bottle of pure IPA. The equivalent concentration in g/ml and ppm for both solutions is 0.008 g/ml and 8 ppm.

EVA Measurement and Two-fold Cross-validation ANN

Four samples were measured with EVA: an empty vial as lab air, 5 ml of normal fNU, 5 ml of K12, and 5 ml of UPEC. Each vial was measured sequentially at a sampling

rate of 10 Hz. Additionally, the measurement sequence was randomized to minimize any history effects. Each sample was measured once in each cycle for ten minutes in a continuous flow and reiterated after all other samples have been measured. Between each vial measurement, the device was also flushed for five minutes to purge off residual VOCs.

With temperature oscillation resulting from the heater voltage waveform over 80 seconds, the six sensors give rise to a total of 120 features. Feature extraction every 80s constitutes one training example for the ANN model for a total of 7 examples per sample per cycle. Upon determining the training and testing sets, both datasets are fed into a backpropagating ANN with two hidden layers of 24 and 9 nodes, respectively, which reports the training and testing results. The accuracy was calculated by dividing the total number of correct predictions over the total number of examples. Second-fold cross-validation was performed by switching the training and testing sets and repeating the analytics. The robustness of the ANN model was assessed with both accuracies.

Preparation of Calibration Standards

Calibration standards were prepared by spiking urine with certain amounts of EtOH and IPA. The final alcohol concentration was calculated using the following equation:

$$C = \frac{X \cdot D \cdot V_{alc}}{V_{urine}} \cdot \frac{10^6\ ppm}{1\ g/ml}$$

where C is the concentration in ppm; X is the dilution factor, which is 1/100; D is the density of the alcohols, which is 0.8 g/ml for both IPA and EtOH; V_{alc} is the volume in µl of the alcohols and V_{urine} the volume in µl of urine.

A preliminary investigation found that the EtOH in UPEC-inoculated urine was approximately 20 to 30 ppm. The concentrations for the EtOH calibrators were thus determined to be 10, 15, 20, 30, 50, 75, and 100 ppm. Approximately 16 milliliters of each concentration were prepared so that they can be split into three batches for subsequent inter-day validation. The exact volume for each concentration was carefully calculated and tabulated in Table 4. After aliquoting 5 ml of each concentration into septa-top vials, each vial was spiked with 31.25 µl of IPA for a final concentration of 50 ppm IPA.

A preliminary investigation found that the EtOH in UPEC-inoculated urine was approximately 20 to 30 ppm. The concentrations for the EtOH calibrators were thus determined to be 10, 15, 20, 30, 50, 75, and 100 ppm. Approximately 16 milliliters of each concentration were prepared so that they can be split into three batches for subsequent inter-day validation. The exact volume for each concentration was carefully calculated and tabulated in Table 4. After aliquoting 5 ml of each concentration into septa-top vials, each vial was spiked with 31.25 µl of IPA for a final concentration of 50 ppm IPA.

Table 4. Serial Dilution of Calibration Standards

Target Concentration (ppm)	Urine Volume (ml)	EtOH Volume (ml)	EtOH Source	Final Volume (ml)
100	47.4	0.6	1/100 Stock	16.0
75	4.0	12.0	100 ppm	16.0
50	20.0	20.0	100 ppm	16.2
30	11.6	17.4	50 ppm	15.7

20	9.6	6.4	50 ppm	16.0
15	8.0	8.0	30 ppm	16.0
10	10.6	5.3	30 ppm	15.9

Preparation of Quality Control Standards

Three Quality Control (QC) standards were prepared at 25 ppm, 60 ppm, and 90 ppm and named QCL, QCM, and QCH, respectively, for low, medium, and high concentration. Ideally, the QCL should be at the maximum of three times higher than the lower limit of quantitation (LLOQ), which is 10 ppm. However, the QC level should not repeat any calibration concentration, so the 25 ppm was determined. They were prepared by directly spiking the urine with the 1/100 ethanol stock. Six QC vials were required, so 32 ml of each QC level was spiked as in Table 5.

Table 5. Preparation Details for QC Standards

QC Name	Concentration (ppm)	EtOH Volume (ml)	Urine Volume (ml)	Final Volume (ml)
QCH	90	0.360	31.64	32
QCM	60	0.240	31.76	32
QCL	25	0.100	31.90	32
LLOQ	10	0.008	31.96	32

The 32-ml QCs were then dispensed into 6 vials at 5 ml per vial and spiked with 31.25 µl of IPA for a final concentration of 50 ppm IPA. The same QC preparation shall

be repeated for each subsequent day of validation. For a full three-day validation, approximately 500 ml of filtered urine is required. The method was validated for its specificity, linearity, intra-day and inter-day inaccuracy and imprecision. The validation method and acceptance criteria were according to Bioanalytical Method Validation, published by the US Food and Drug Administration [20].

GC-MS Parameters and Conditions

The GC-MS used was an Agilent 6890 GC system equipped with HP 5973 Mass Selective Detector. Detection was done using a quadrupole detector with an electron impact source. The analytical run was performed on a DB ALC column with 30-m length, 0.32 internal diameter, and 1.80 film thickness. Inlet temperature was set at 100°C. The oven temperature was initially held for two minutes at 40°C then ramped for 25°C/min until 250°C and held for another two minutes, and the total running time was 26 minutes under the scanning mode. Under the selected ion monitoring (SIM) mode, inlet temperature was set at 100oC and the column temperature at 40°C without temperature ramping. The column flow was set at 6 mL/min with a total runtime of six minutes. The split mode was used with a split ratio of 25:1. Helium was used as a carrier gas with a flow rate of 0.9 mL/min. Isopropyl alcohol was used as an internal standard (ISTD). A Hamilton 1005SL 5-ml gas-tight syringe was used to withdraw and inject the headspace samples into the GC-MS manually.

Before the injection, the sample was placed in a heat block that had been heated to approximately 80°C and kept incubating for ten minutes to increase headspace VOCs (S1 Fig). A metal blockade was placed on top of the vial cap to prevent accidental touching

of the syringe tip and the liquid. Five milliliters of the headspace sample was injected into GC-MS under scanning mode as described previously to determine the compound of interest. After injection, the syringe was thoroughly cleaned with water and dried with an air gun.

Assessment of Sample Stability after EVA Measurement

Sample evaporation becomes an issue during EVA measurement due to continuous suction of the headspace volume. It was observed that the sample volume got reduced over time. Therefore, the newly developed method was used to quantitate the stability of ethanol content in K12-inoculated urine during measurement.

Due to the destructive nature of the GC-MS assay, multiple spiked samples were prepared to be measured at different time points (S2 Fig). A stock of 25-ppm ethanol-spiked urine stock was prepared and split into nine vials. One vial was quantitated with the GC-MS to confirm the initial concentration. The remaining eight vials were divided into two groups: one group was measured by the EVA, and the other group was not (control). One vial from each group was quantitated by GC-MS after each EVA measurement cycle of ten minutes.

Results and Discussion

Specificity

The mass over charge number ratios (m/z) 31 and 45 for ethanol and the m/z 45 and 43 for isopropyl alcohol were selected based on pre-scanning of ethanol and IPA.

Blank urine sample, blank urine sample spiked with the internal standard, and blank urine sample spiked with 10 ppm EtOH and 50 ppm ISTD were prepared and injected into GC-MS. Two separate peaks were observed for EtOH and ISTD (Fig 3). In particular, ethanol was detected at 4.38 min and IPA at 5.45 min. No significant interfering peaks were found at the retention times at which ethanol and IPA appears. The signal to noise ratios for both drugs was greater than 10. The results demonstrate the adequacy of the method for the specificity of the compounds involved.

Fig 3. Chromatograms of (a) blank urine, (b) urine sample spiked with 50 ppm IPA, and (c) urine sample spiked with 10 ppm ethanol and 50 ppm IPA.

Linearity

A calibration curve was plotted for each validation day for the ratio of EtOH to ISTD response against the concentrations of ethanol (Fig 4). The linearity was observed for all calibration curves performed during method validation with all R^2 values above 0.990 (Table 6).

Fig 4. A representative standard curve. The y-axis plots the response ratio between the internal standard and the analyte.

Table 6. Standard Curve Equations and Their Coefficients of Determination

Validation assay	Linear equation	R^2
Day 1	y = 0.0064x + 0.0096	0.9957

Day 2	y = 0.0065x + 0.0068	0.9916
Day 3	y = 0.0063x + 0.0134	0.9916

Within-Assay Reproducibility

The mean concentration from six replicates of each QC level was calculated, along with their standard deviation and coefficient of variation (Table 7). The mean inaccuracy was calculated by averaging the percent difference between each data point and the mean value. All inaccuracy values were within acceptable ranges according to US FDA guidelines (20% or less for LLOQ and 15% or less for other QCs).

Table 7. Within-Assay Coefficients of Variation and Mean Inaccuracies

Batch number	QC	Nominal conc. (ppm)	Mean (n=6) (ppm)	SD (ppm)	CV (%)	Mean inaccuracy (%)
1	LLOQ	10	8.50	0.83	9.76	15.00
	QCL	25	24.24	1.31	5.40	4.9
	QCM	60	61.59	3.19	5.18	4.74
	QCH	90	98.01	4.77	4.87	9.01
2	LLOQ	10	8.70	1.07	12.30	15.62
	QCL	25	22.98	2.44	10.62	11.89
	QCM	60	55.68	7.47	13.42	12.92
	QCH	90	87.47	5.63	6.44	5.49
3	LLOQ	10	11.06	0.97	8.77	10.63

QCL	25	25.07	3.30	13.16	8.80
QCM	60	62.38	3.45	5.53	4.79
QCH	90	89.87	4.63	5.15	4.25

Between-Assay Repeatability

The between-assay repeatability was assessed by calculating the mean, the standard deviation, the CV, and the mean inaccuracy across all 18 samples from the three batches. All mean inaccuracy values were within acceptable ranges according to FDA guidelines (Table 8).

Table 8. Between-Assay Coefficients of Variation and Mean Inaccuracies

QC	Nominal conc. (ppm)	Mean (n=18) (ppm)	SD (ppm)	CV (%)	Mean inaccuracy (%)
LLOQ	10	9.42	1.5	15.92	13.75
QCL	25	24.1	2.5	10.37	8.56
QCM	60	59.88	5.69	9.50	7.48
QCH	90	91.79	6.62	7.21	6.25

Quantitation of Ethanol in *E. coli*-inoculated Urine

There have already been numerous studies on the e-nose application in detecting and differentiating the causative pathogeni[illegible]pecies. Interestingly, these studies looked at the VOCs of bacteria in a nutrient agar or gelified urine instead of directly measuring

the headspace of the urine sample. Presumably, the solidified form could minimize the evaporation of background components since the presence of water molecules and volatile nutrient broth components could have introduced variability to the sensor system. It is thus important to demonstrate the differentiation capability of an e-nose directly within the headspace of liquid samples for better representation as a point-of-care device. Therefore, the GC-MS method was used to measure samples of K12-inoculated and UPEC-inoculated urine in tandem with an artificial neural network classification of odor data collected by the EVA.

The GC-MS quantitation resulted in 31.33 ppm of ethanol in UPEC-inoculated urine and 18.00 ppm in K12-inoculated urine. Besides the two abovementioned labels, the EVA also measured lab air and normal urine. The dataset was split in half for the training and testing set and evaluated with two-fold cross-validation. The classification resulted in 100% accuracy for both validations, which was determined by taking the ratio between the number of correctly predicted examples over the total number of examples. The VOC fingerprints are visualized by plotting the log of the electrical resistance across all features (Fig 5). The results thus highlighted the potential of EVA to distinguish bacterial strains directly on liquid samples.

Fig 5. Visualization of VOC fingerprints shows different patterns in electrical resistance across the features.

These results demonstrated that UPEC and K12 *E. coli* produced distinguishable levels of ethanol, thus causing different VOC fingerprints. The four color-coded labels

clearly show different patterns from one another when taking all feature responses as a whole. Without extensive scans, it is not known whether the two strains also have distinct levels of other VOCs. However, the difference in compound concentration enables the differentiation of these two strains of the same species. The GC-MS method allows researchers to directly study the VOC profiles in inoculated urine and aids in decision-making regarding establishing a better measurement protocol and choice of training data for e-nose application.

Sample Instability and Consideration for EVA Measurement Time

The concentration of ethanol-spiked urine was quantitated after every EVA cycle for four cycles. Adhering to the FDA acceptance range, a percent change of 15% or more was deemed significant. Fig 6 shows that the concentration of ethanol diminished significantly (more than 15%) as early as after the third cycle, which is equivalent to 30 minutes of measurement per vial.

Fig 6. Percent change in ethanol concentration after each EVA measurement cycle.

Next, a new classification was performed using only three labels of lab air, normal urine, and K12. Data were collected up to 15 cycles each day for two days. The data from the first three cycles were used as a training set to test every subsequent set of three cycles. The results in Table 9 indicate that accurate classification was possible up to nine

cycles. Therefore, there are two thresholds for measurement cut-off: the first three cycles and the first nine cycles.

Table 9. Classification Accuracies from Training with the First Three Cycles

**Tested on:**	Cycles 4-6	Cycles 7-9	Cycles 10-12	Cycles 13-15
**Average accuracy:**	95.70%	98.20%	55.50%	37.10%

Between-day classifications were used to evaluate the two cut-offs, and the results are plotted in Fig 8. Training with the first 30 minutes (three cycles) of data gives better accuracies of 97.0% and 73.7% compared to training with the first 90 minutes of data.

Fig 7. Cross-validation accuracies between days for 90-min and 30-min measurements.

Thus, sample instability was induced after 30 minutes of measurement with an e-nose. Specifically, the ethanol concentration was reduced by more than 15%, which is significant according to the FDA guidelines. It was further demonstrated that the ANN model gave superior classification accuracies when using only data from the first 30 minutes of measurement. The result is an example of machine learning basics: data quality is more important than data quantity. The inclusion of the data from unstable samples was detrimental to the classification model. The GC-MS method was thus proved useful for determining the measurement cut-off time.

Since e-noses typically output electrical resistances, which result from the collective interaction of the whole VOC profile with the sensors, they lack the credibility to aid in decision making regarding quality control of the data. Furthermore, we recognized that the VOC profiles in a real patient-derived UTI samples are likely more complicated. Future studies should carefully evaluate the possibility of sample degradation. The same headspace analysis should be considered in every future experimental design to determine the ground truth that is the concentration of analytes before proceeding to use the collected data. By characterizing the sample stability during measurement, one can safely reduce variability in the data due to the very act of measurement itself while being able to incorporate background compounds as part of the VOC profile.

Conclusion

In this paper, we demonstrated the validation and quantitation data of ethanol in the headspace of the urine samples inoculated with *E. coli*. To our knowledge, this is the first paper describing the validation and quantitation of alcohol release from the bacteria by using inoculated urine samples. The method validation was fulfilled all the criteria as outline in the Bioanalytical Method Validation protocol published by the US FDA. The method was successfully applied for the ethanol measurement in samples of K12 and UPEC-inoculated urine in order to improve the outcome of VOC measurement by the e-nose developed by IBM. The main interest of using this method was to characterize *E. coli*-inoculated urine samples that are prepared for training an electronic nose to detect urinary tract infection and differentiate between different causative agents. By using the new method, it was shown that different strains of *E. coli* could produce different levels of

ethanol concentration, making it possible to differentiate between them based on distinct VOC fingerprints. The quantitation also revealed that e-nose measurement could affect sample stability over time. Therefore, the cut-off time for future measurement should be wisely determined to avoid the collection of unusable data due to a reduction in compound concentration. The GC-MS would continue to be a useful tool for characterizing samples for building a useful training library for UTI detection directly through liquid samples without extra preparation steps, thus enabling future real-time and point-of-care diagnosis of UTI.

Acknowledgments

The authors gratefully acknowledge the financial support of IBM Almaden Research Center, San Jose, CA, and the Ministry of Higher Education Malaysia for the supporting research scholar working on this project.

References

1. Kwak J, Preti G. Volatile Disease Biomarkers in Breath: A Critique. Curr Pharm Biotechnol. 2011;12: 1067–1074. doi:10.2174/138920111795909050
2. Meinardi S, Jin KB, Barletta B, Blake DR, Vaziri ND. Exhaled breath and fecal volatile organic biomarkers of chronic kidney disease. Biochim Biophys Acta - Gen Subj. 2013;1830: 2531–2537. doi:10.1016/j.bbagen.2012.12.006
3. Zhang S, Liu L, Steffen D, Ye T, Raftery D, Ms GC, et al. Metabolic profiling of gender : Headspace-SPME / GC – MS and 1 H NMR analysis of urine. 2012;

323–334. doi:10.1007/s11306-011-0315-2

4. Roine A, Saviauk T, Kumpulainen P, Karjalainen M, Tuokko A, Aittoniemi J, et al. Rapid and accurate detection of urinary pathogens by mobile IMS-based electronic nose: A proof-of-principle study. PLoS One. 2014;9. doi:10.1371/journal.pone.0114279
5. Bos LDJ, Sterk PJ, Schultz MJ. Volatile Metabolites of Pathogens : A Systematic Review. 2013;9: 1–8. doi:10.1371/journal.ppat.1003311
6. Storer MK, Hibbard-melles K, Davis B, Scotter J. Detection of volatile compounds produced by microbial growth in urine by selected ion fl ow tube mass spectrometry (SIFT-MS). J Microbiol Methods. 2011;87: 111–113. doi:10.1016/j.mimet.2011.06.012
7. Craven MA. Bacteria classification with an electronic nose employing artificial neural networks. 1997.
8. Gibson TD, Prosser O, Hulbert JN, Marshall RW, Corcoran P, Lowery P, et al. Detection and simultaneous identification of microorganisms from headspace samples using an electronic nose. Sensors Actuators, B Chem. 1997. doi:10.1016/S0925-4005(97)00235-9
9. Gardner JW, Shin HW, Hines EL. Electronic nose system to diagnose illness. Sensors Actuators, B Chem. 2000;70: 19–24. doi:10.1016/S0925-4005(00)00548-7
10. Pavlou AK, Magan N, McNulty C, Jones J, Sharp D, Brown J, et al. Use of an electronic nose system for diagnoses of urinary tract infections. Biosens Bioelectron. 2002;17: 893–899. doi:S095656630200787 [pii]

11. Asimakopoulos AD, Del Fabbro D, Miano R, Santonico M, Capuano R, Pennazza G, et al. Prostate cancer diagnosis through electronic nose in the urine headspace setting: a pilot study. Prostate Cancer Prostatic Dis. 2014;17: 206–211. doi:10.1038/pcan.2014.11
12. Flores-Meireles A, Walker J, Caparon M, Hultgren S. Urinary tract infections: epidemiology, mechanisms of infection and treatment options. Nat Rev Microbiol. 2015;13: 269–284. doi:10.1038/nrmicro3432.Urinary
13. Khodadadi M, Pourfarzam M. A review of strategies for untargeted urinary metabolomic analysis using gas chromatography–mass spectrometry. Metabolomics. 2020;16: 66. doi:10.1007/s11306-020-01687-x
14. Tangerman A. Highly sensitive gas chromatographic analysis of ethanol in whole blood, serum, urine, and fecal supernatants by the direct injection method. Clin Chem. 1997;43: 1003–1009. doi:10.1093/clinchem/43.6.1003
15. Mihretu LD, Gebru AG, Mekonnen KN, Asgedom AG, Desta YH. Determination of ethanol in blood using headspace gas chromatography with flameionization detector (HS-GC-FID): Validation of a method. Fan J-P, editor. Cogent Chem. 2020;6: 1760187. doi:10.1080/23312009.2020.1760187
16. Chun H-J, Poklis JL, Poklis A, Wolf CE. Development and Validation of a Method for Alcohol Analysis in Brain Tissue by Headspace Gas Chromatography with Flame Ionization Detector. J Anal Toxicol. 2016/08/03. 2016;40: 653–658. doi:10.1093/jat/bkw075
17. Xiao H, He L, Tong R, Yu J, Chen L, Zou J, et al. Rapid and Sensitive Headspace Gas Chromatography–Mass Spectrometry Method for the Analysis of Ethanol in

the Whole Blood. J Clin Lab Anal. 2014;28: 386–390. doi:10.1002/jcla.21698

18. Kristoffersen L, Stormyhr L-E, Smith-Kielland A. Headspace gas chromatographic determination of ethanol: The use of factorial design to study effects of blood storage and headspace conditions on ethanol stability and acetaldehyde formation in whole blood and plasma. Forensic Sci Int. 2006;161: 151–157. doi:https://doi.org/10.1016/j.forsciint.2006.03.034
19. Smith D, Španěl P, Holland TA, Al Singari W, Elder JB. Selected ion flow tube mass spectrometry of urine headspace. Rapid Commun Mass Spectrom. 1999;13: 724–729. doi:10.1002/(SICI)1097-0231(19990430)13:8<724::AID-RCM548>3.0.CO;2-E
20. FDA F and DA. Bioanalytical Method Validation Guidance. Food Drug Adm. 2018;1043: 25.

Supporting information

S1 Fig. Headspace sample being withdrawn from urine vial heated in a heat block. The real temperature was measured in an adjacent water vial by a digital thermometer. Inset: Urine sample in a septa-top vial for headspace analysis.

S2 Fig. A schematic of urine sample stability test with GC-MS

Figure 1

Click here to access/download;Figure;Fig1.tif

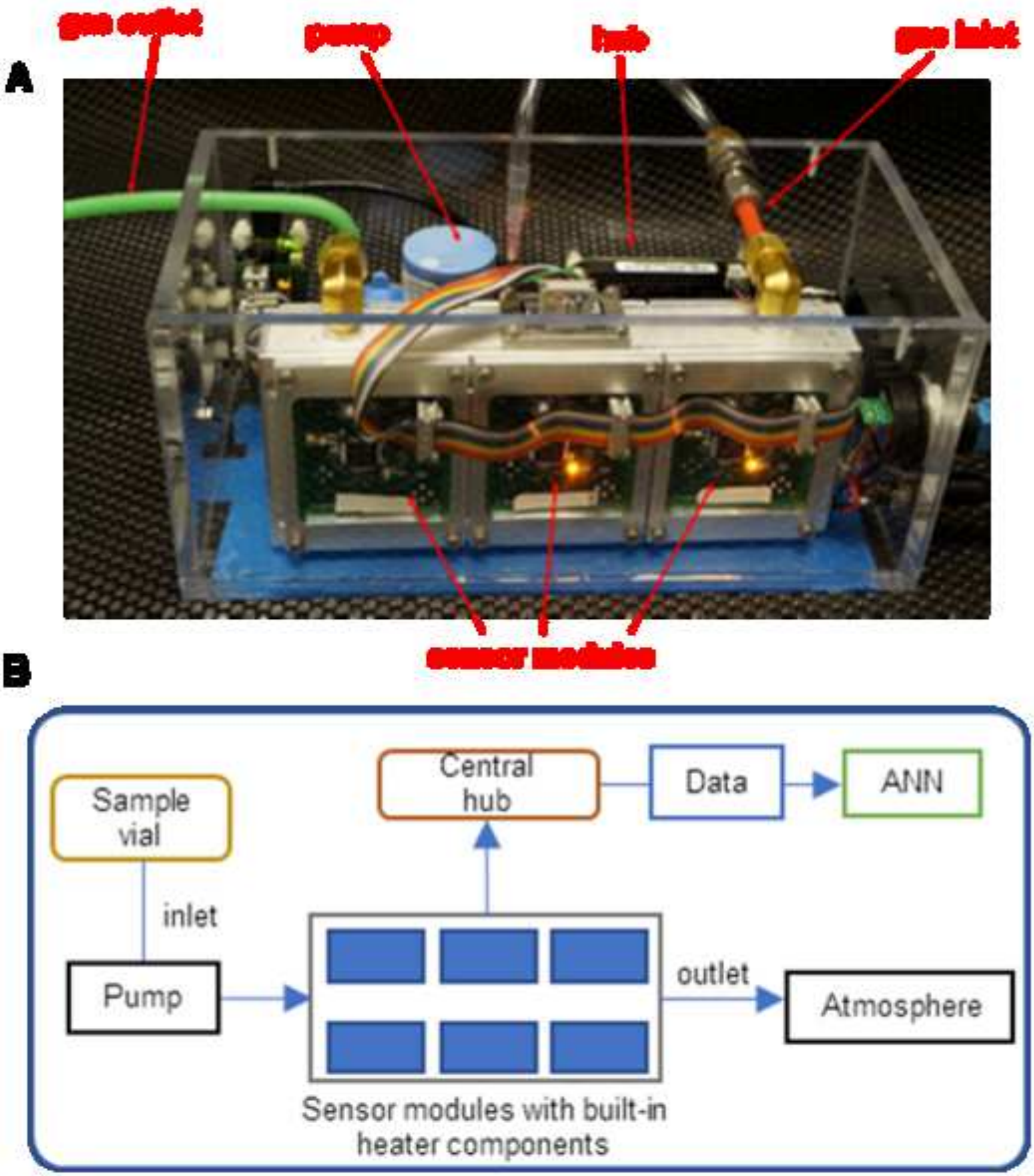

Figure 2

Click here to access/download;Figure;Fig2.tif

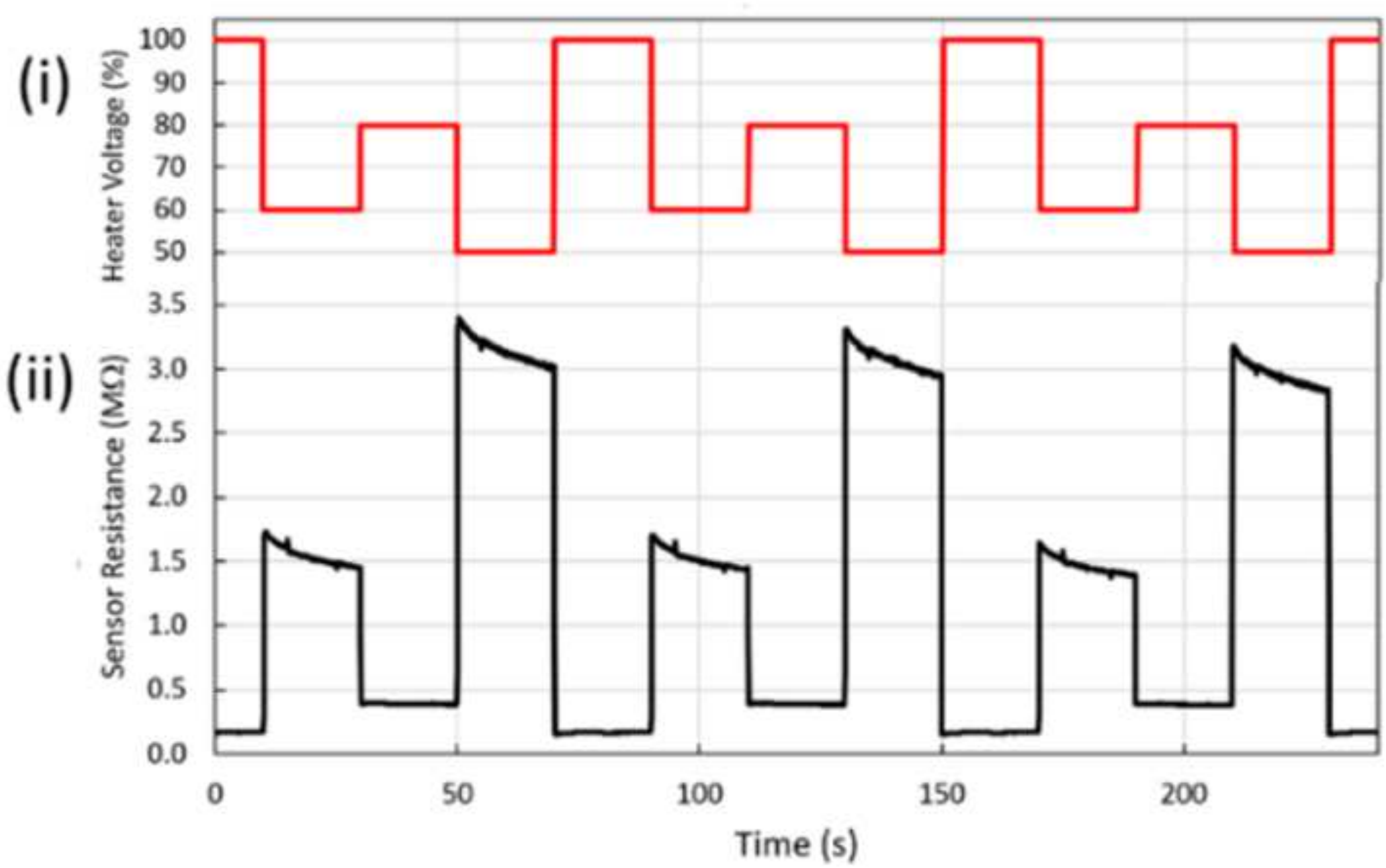

Figure 3

Click here to access/download;Figure;Fig3.tif

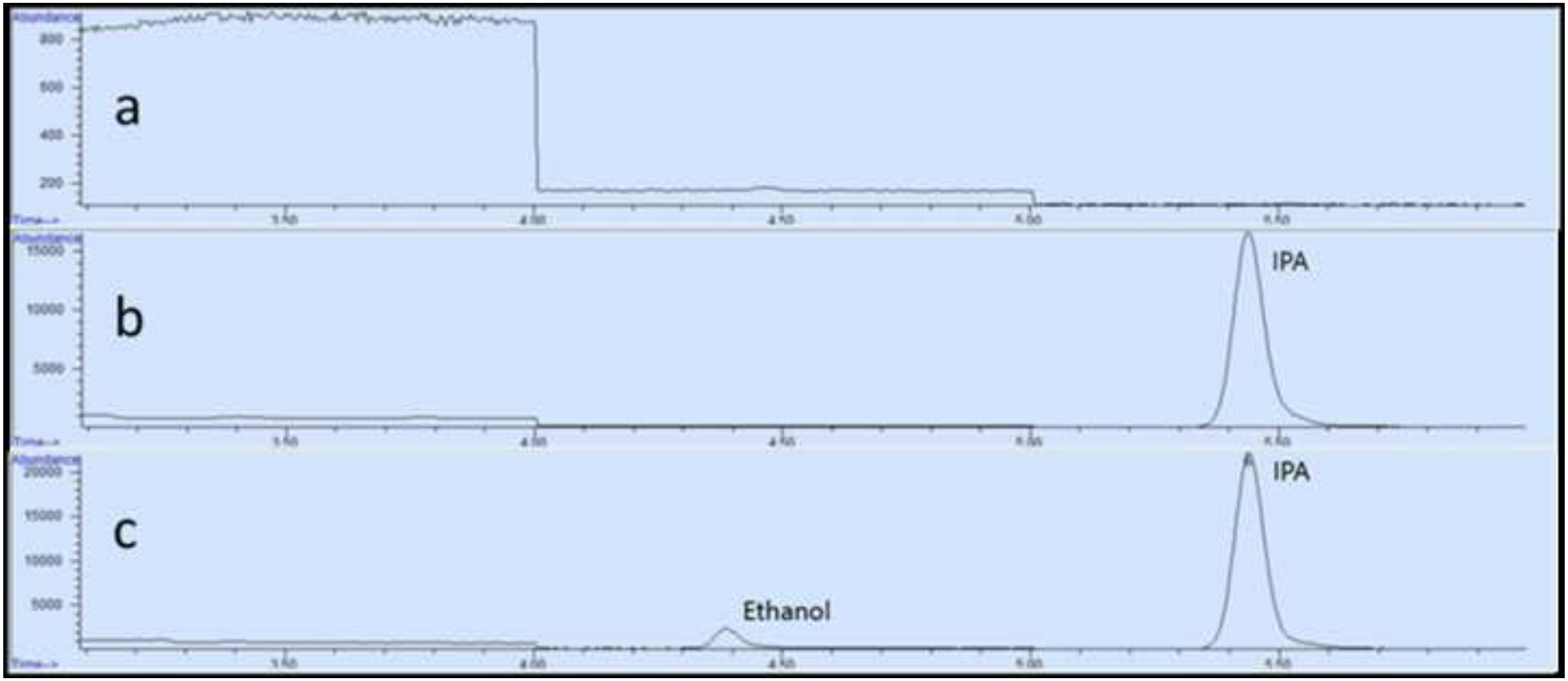

Figure 4

Click here to access/download;Figure;Fig4.tif

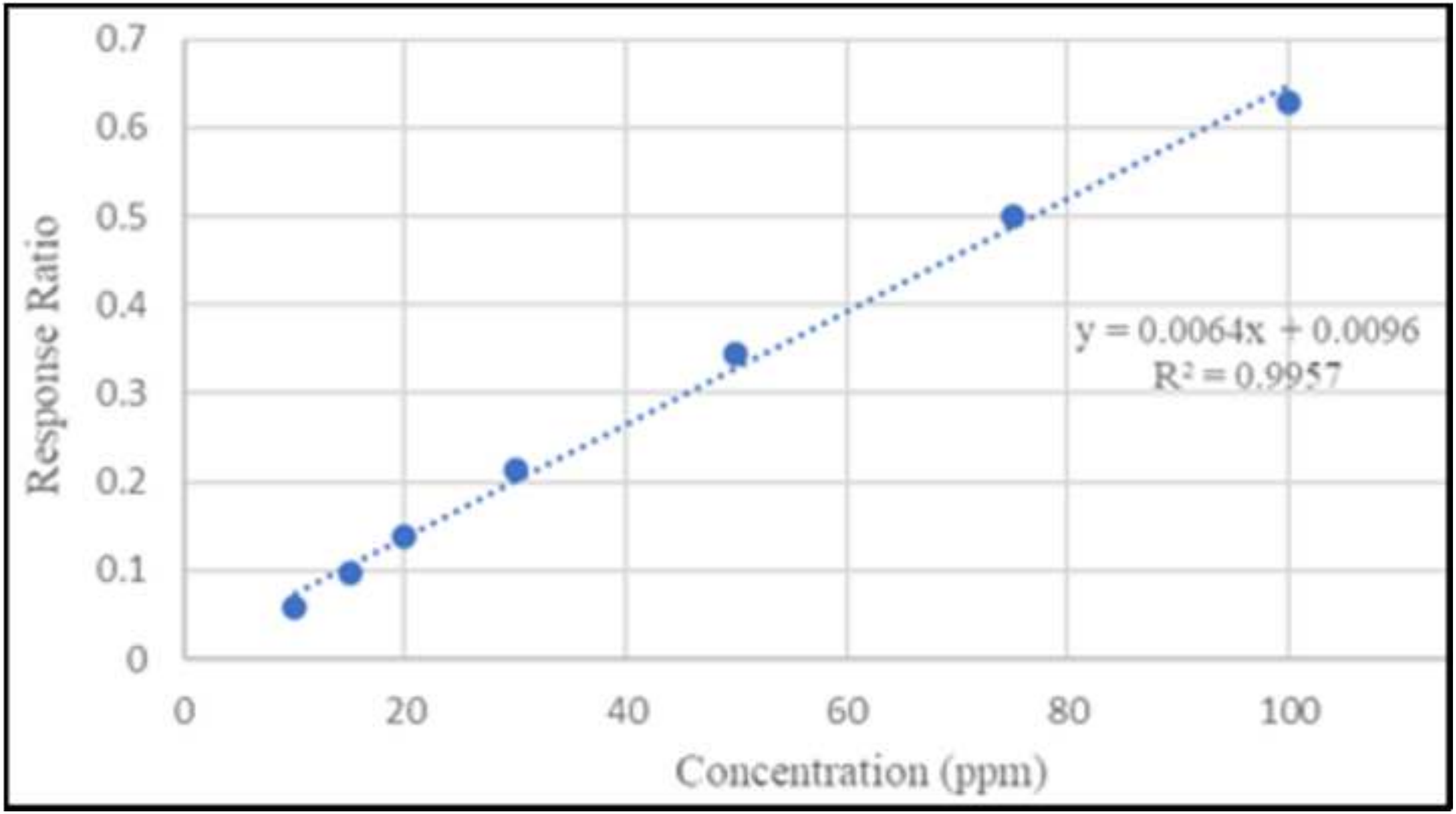

Figure 5

Click here to access/download;Figure;Fig5.tif

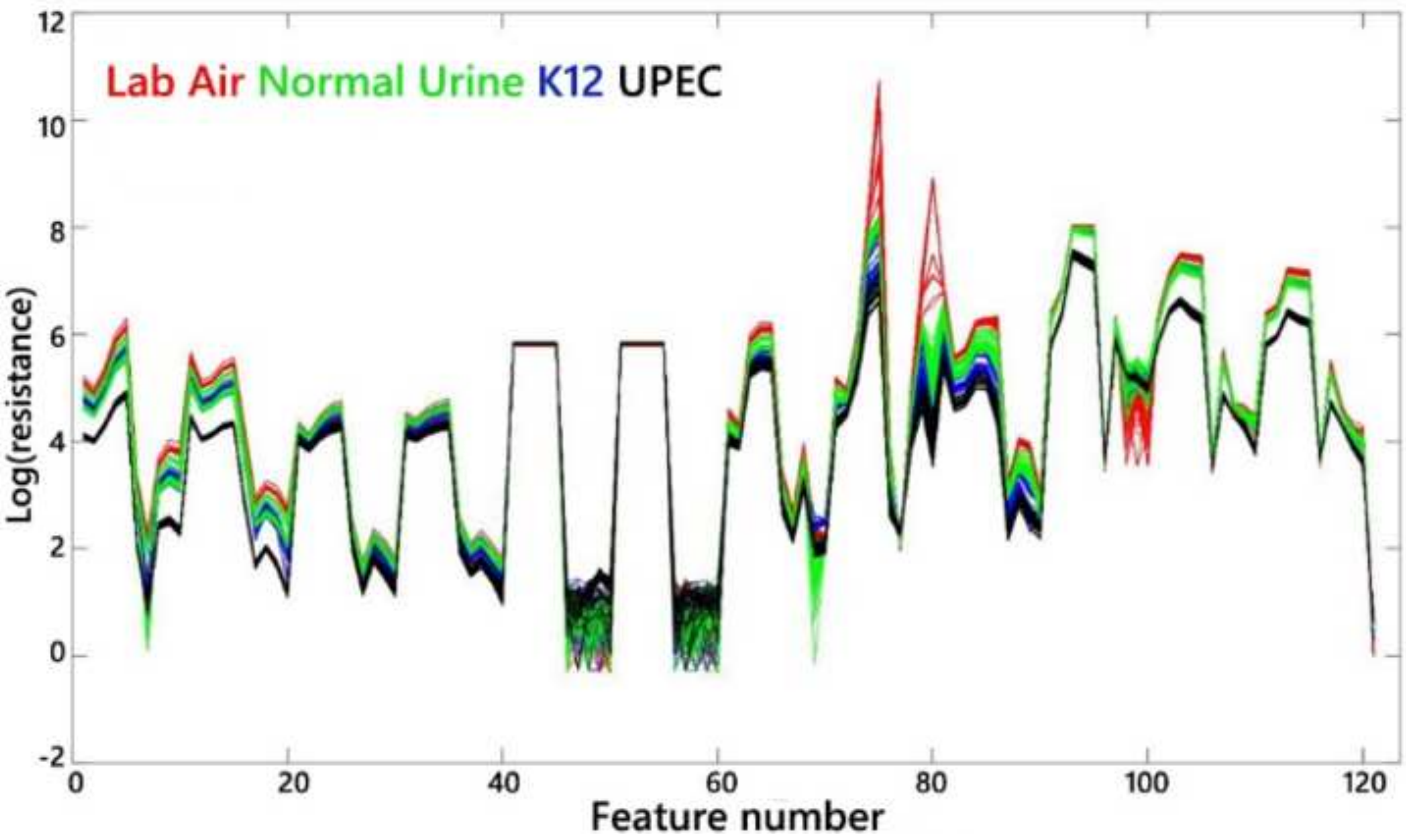

Figure 6

Click here to access/download;Figure;Fig6.tif

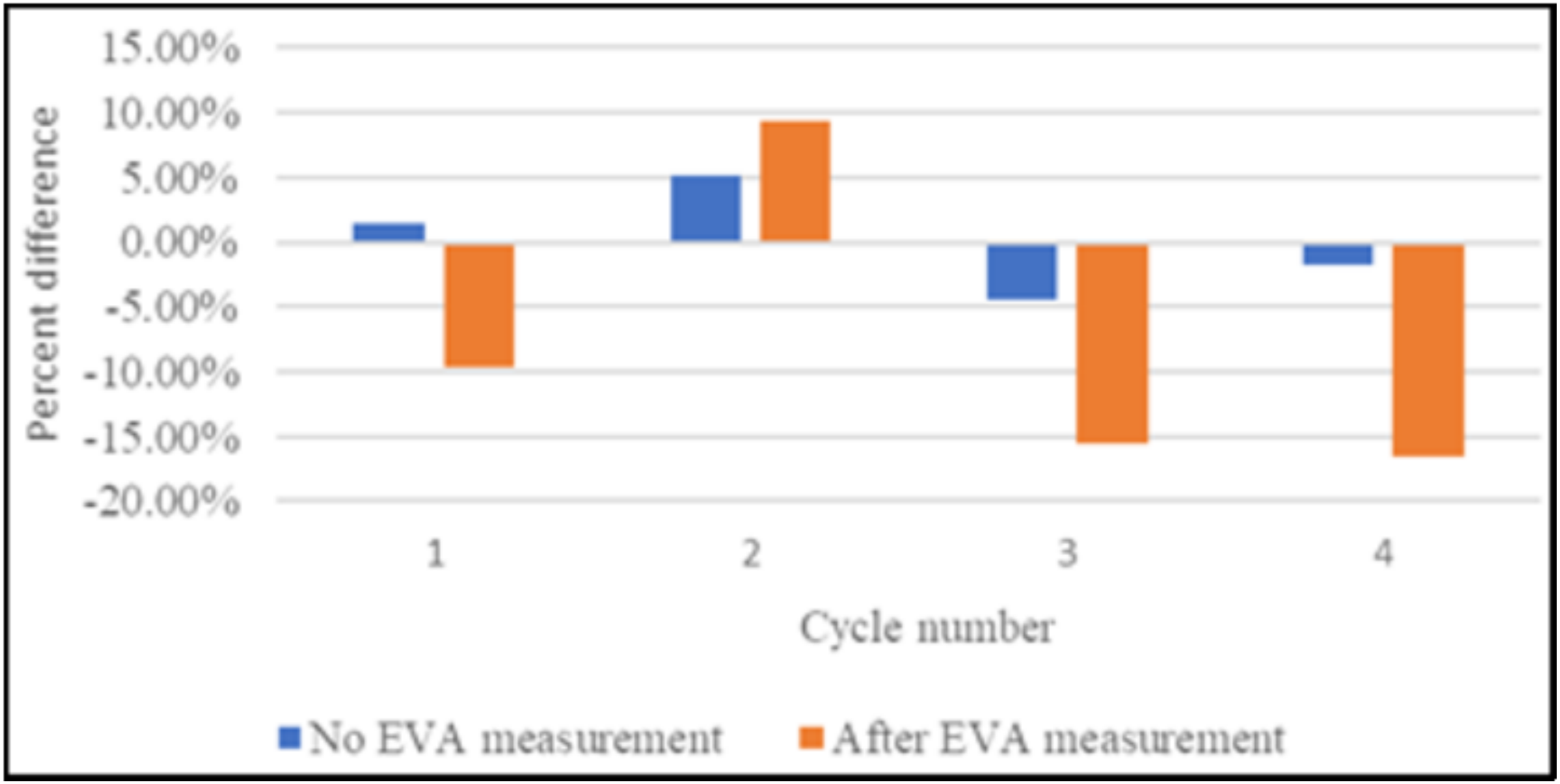

Figure 7

Click here to access/download;Figure;Fig7.tif

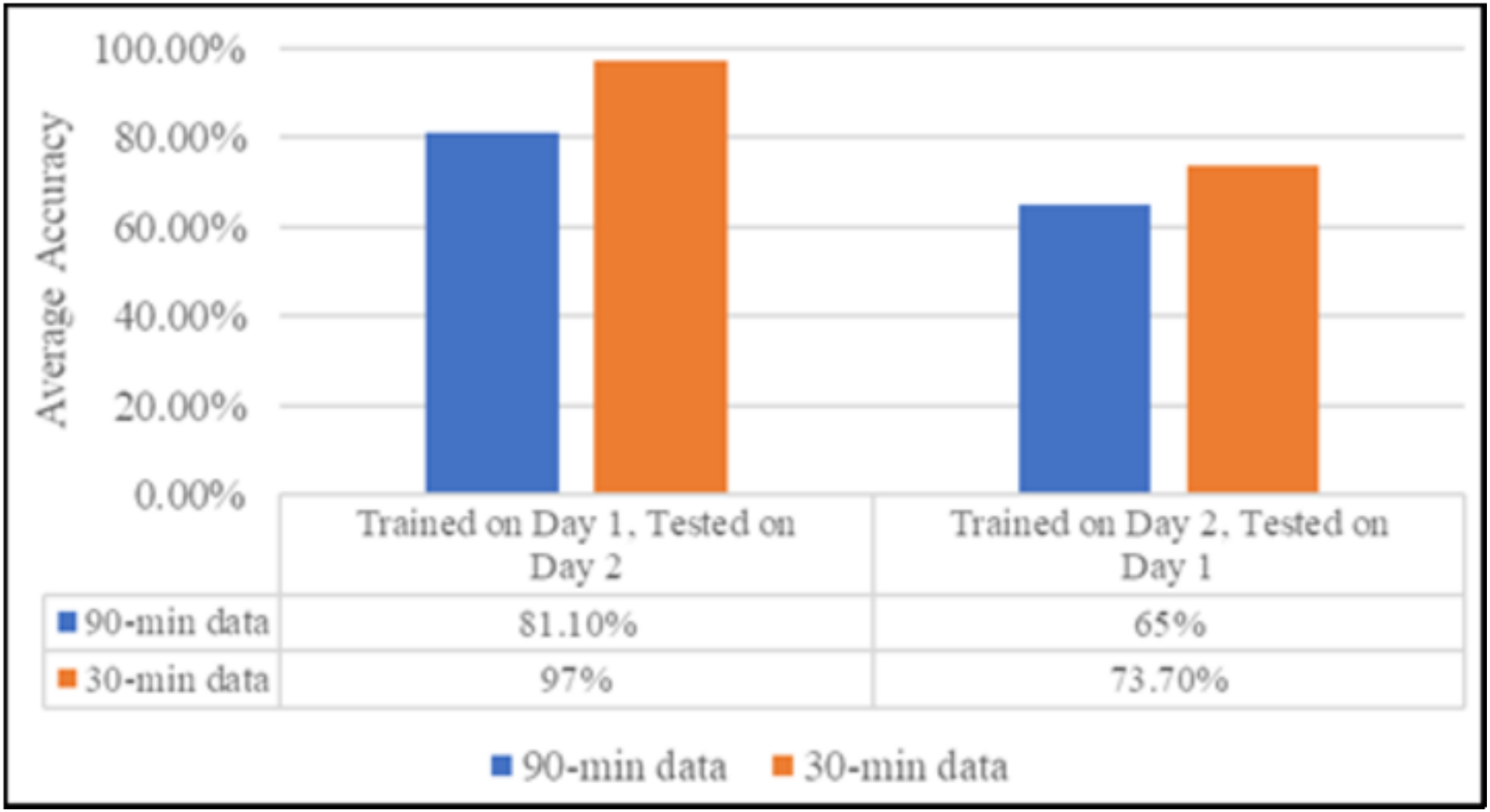

Sup Fig 1

Click here to access/download
Supporting Information
S1 Fig.tif

Sup Fig 2

Click here to access/download
Supporting Information
S2 Fig.tif
