# Supplementary material for: Quantitation of ethanol in UTI assay for volatile organic compound detection by electronic nose using the validated headspace GC-MS method
Source: PLoS One. 2022 Oct 6;17(10):e0275517. doi: 10.1371/journal.pone.0275517 (PMC9536638; doi:10.1371/journal.pone.0275517)
Supplement: Attachment — Submitted filename: Response to Reviewers_July2022review.docx [file pone.0275517.s006.docx]

**RESPONSE TO REVIEWERS (PONE-D-21-22287)**

We are delighted to receive positive comments from reviewers # 1 and 5 and acknowledge concerns raised by reviewer #2. We have addressed all the comments in our revised manuscript, and the response details are as follows. We are happy to answer further concerns and questions from the reviewers.

**Reviewer 1:**

**"I was excited to see your new version. All my previous comments have been addressed. The changes to the introduction made it more relevant and interesting to read. I hope your future research projects will go well. I am looking forward to seeing more IBM EVA use."**

We appreciate all the positive comments from Reviewer 1 and grateful for your careful evaluation of our manuscript.

**Line 143 "in" should be added: "results (in) under two minutes"**

Please note that the line numbers hereafter refer to the no-mark-up version of our new revision.

Line 148: We corrected it.

**Line 303 there are two full stops after mL/min.**

I could not identify the mistake. The manuscript had only one full stop after mL/min at line 301.

**Line 370 would be better if "there are" was used : "[...] (there are) numerous studies on the e-nose application [...]".**

Thank you for your suggestion. We made the change at line 379.

**Reviewer 2**

**"The paper is much improved, but there are a number of points outstanding and a number of answers were not added to the manuscript. Below are some more specific comments."**
**Line 97: I would add a reference to the original paper on this by Dodd & Persaud in Nature from the early 1980s.**

Please note that the line numbers hereafter refer to the no-mark-up version of our new revision.

Line 87: We added the paper, which was an excellent reference to the origin of e-nose technology.

**Line 106: I am surprised there haven't been more papers and more recent ones. These are very old. Can this table be updated?**

Line 95: We updated the table with a few more recent references. However, to our surprise, not many new papers specifically looked at the urine-based classification of pathogens in the context of urinary disease. We reflected this in the paragraphs following table 1.

**Line 113: PCA is not a classifier, so this will need to be altered to make sense.**

We removed that line and modified the paragraph (not lines 97-108).

**Line 133: I would add the paper by Estfani or urine storage by FAIMS (I might have the name wrong). It showed samples were good for 9months+ at -80C. I am also sure that some of Dutch group have done work on storage at room temperature. Might be worth adding an extra reference or two here.**
Line 114: We appreciate your helpful comment. Esfahani *et al.* (2016 & 2018) are good papers to be included as it shows that despite storing at -80^o^C, storage time still significantly affects classification accuracy. They suggested samples were good within 9 months (in the 2016 paper) and that performing urine analysis within a year of storage resulted in better accuracy than including all samples within four years. Their 2018 study only considered two labels (healthy vs. diabetes). An even shorter storage time could be recommended for a more complex analysis involving more labels.

**Line 153: Usually ethanol is undertaken using a single optical gas sensor – for example they are used in breath ethanol testing. This should be included in the description as they are pretty common.**
Line 138: We updated this line to add the breath analyzer as an example of an ethanol quantification system.

**Line 162: I am pretty sure the EVA has been reported before. I would rephase this sentence.**

Line 146 We have introduced the EVA before in a conference proceeding (*Adebiyi et al.* 2019). Therefore, we rewrote the sentence as "In this paper, we used the Electronic Volatile Analyzer (EVA)…" and cited this reference.

**Line 181: So, why not just use the ethanol sensor are be done?**
Line165: Ethanol sensors alone will not create enough differential response to discriminate many different VOC profiles. We mainly measured ethanol as an indication of sample stability because we inoculated urine with ethanol-producing bacteria (E. coli). However, a robust e-nose requires a combination of sensors responsive to various compounds. We revised this line to reflect these points.

**Line 461: I am more interested in the stability of your instrument and the sensors over the sample. I may not have got to it yet, but how long did the calibration last?**
Line 434: We believe we shared this in our previous review, but the stability of our sensors over the sample period was reproducible from measurement to measurement. From a cold-start of the instrument, we ran air (our carrier gas) through the chamber for sixty minutes, before we moved on to the measurement samples. Between each 15 minute measurement, we ran air for five minutes to purge any residual VOCs.

**Line 512: I would like to see a section on limitations of the study and of the use of eNoses for this purpose. For example, drift (of the various forms) is a good example. Also, how well the system will be able to cope with variations in urine. With UTIs urine can be almost clear and others the consistency of porridge.**
Line 481: We added a paragraph on the limitations of the study. We think the consistency of urine is not a problem per se but what VOC profile they will produce is the determining factor. Obviously, cloudy vs clear urine may have different VOCs in them, some may not be even related to UTI. Therefore, screening for prevalent compounds in UTI vs healthy controls is important and optimizing sensor choice that targets these compounds. Sensor drift is a notable limitation of electronic noses given the variability of the MoX sensors in undergoing these oxidation reactions over time. Sensor drifting can be compensated by a multitude of mathematical techniques (newly included in the paper) such as baseline correction and the like, but our instrument is designed for real-time use”in the wild”, so this choice of calibration was a design choice meant to be as robust as possible to accommodate mostly operator-free scenarios.

**I would still like to see a PCA from this dataset in the paper and a loading plot for the PCA. Unless the authors are saying this is the same dataset, then adding a new PCA is appropriate.**
Our dataset is the same as the last review. We have tried PCA but found ANNs more suitable to capture the drift and variability in the model for our use-case and setup (which we also described previously). We are happy to provide the reviewer with a PCA for their reference, but a study by itself can be conducted on the choice of mathematical approaches suitable. Given the scope of this paper was mainly to quantify the levels of ethanol in our samples with the validated GC-MS method, we did not go into all those details. An example of a reference related to these mathematical methods with the PCA loadings can be found in Figure 3 of this reference [30] in our paper, which provides these details.

Adebiyi, A., Than, N., Swaminathan, S., Abdi, M., Bowers, A. N., Fasoli, A., ... & Bozano, L. (2020, January). Rapid Strain Differentiation of E. coli-inoculated Urine Using Olfactory-based Smart Sensors. In *SEIA'2019 Conference Proceedings* (p. 307).

**The length per test is still significant. It just needs to be added as a limitation of the study as there is no way to re-do the experiments. You can get electronic noses to respond in seconds, just not how you tested it.**
We have addressed it in the limitation paragraph. However, we would like to add that this length (15 minutes) signifies the measurement stage to train the model. At inference, classification takes 80 seconds.

**From the previous points (and I state above) I can just go purchase a cheap ethanol sensor, so the reason for needing to use an electronic nose for this purpose needs to be explained in more detail. I could even get a cheap MOX sensor and make it a lot more specific to ethanol…you just need to justify better why you would use an expensive eNose over a cheap single sensor.**
Line 475: We appreciate that cheap ethanol sensors are ubiquitous and finding a correlation between the quantity of ethanol and E. coli strain type could potentially aid in strain differentiation. In some ways, this is what we sought to explore by using the validated GC-MS method to determine if the change in ethanol is mainly due to the degradation of the sample vs the ethanol secreted by the given E. coli strain at incubation. It could be more difficult for a cheap ethanol sensor to tell if it is UPEC E. coli after the sample had been sitting for three days than K12 after two. If we did seek to use the cheap ethanol sensors, we would stil have to validate it, but we could add that as a point. Speaking to the justification of the EVA as a device, we are seeking to build a library of different types of bacteria, not just speficially those that are related to ethanol, it just happens that ethanol is the main VoC in this use-case.

**Details of the EVA need to make it to the paper (I didn't see the additions). Also add details of the optimisation and not just the patent – to the paper…**
As we mentioned previously, the scope of this paper is intended to look at the quantification of the ethanol method, and not to provide a deep dive into the hardware optimization of the EVA, which could be its own study. We are happy to reference this patent in paper, but believe that adding all the details will take away from the focus of this study. However, we briefly summarize the operational basis of EVA on page 10, which we believe is appropriate to reflect the focus of this paper.

**Please add all feature extraction methods to the paper. Also software used etc.**

Line 247: We have added this to the paper.

**Reviewer #5:**

**Though the manuscript is well designed and presented, I am doubtful regarding the applicability of the developed method. The manuscript can be considered for publication provided that comments and suggestions given by the other reviewers are fully addressed.**

We are thankful for the positive comment. We believe other researchers in the field can adapt the developed method to characterize the content in their e-nose samples. Not only for ethanol but other compounds can also be quantified by modifying the validated method.
